# Rate and Modifiable Predictors of 30-Day Readmission in Patients with Acute Respiratory Distress Syndrome in the United States

**DOI:** 10.7759/cureus.8922

**Published:** 2020-06-30

**Authors:** Harshil Shah, Uvesh Mansuri, Sukrut Pagad, Reshmi Adupa, Jagmeet Singh, Khin Tun, Chail Shah, Solomon Tuonuur, Priyal J Shah, Mir Z Ali Khan, Gurjot S Grewal, Ruchir Goswami, Shantanu Solanki

**Affiliations:** 1 Internal Medicine, Independent Researcher, Sayre, USA; 2 Medicine, MedStar Union Memorial Hospital, Baltimore, USA; 3 Internal Medicine, Larkin Community Hospital, Hialeah, USA; 4 Internal Medicine, Garden City Hospital, Garden City, USA; 5 Nephrology, Geisinger Commonwealth School of Medicine, Scranton, USA; 6 Pediatrics, Independent Researcher, Yangon, MMR; 7 Internal Medicine, Brooklyn Cancer Care, Brooklyn, USA; 8 Internal Medicine, Mahatma Gandhi Medical College and Research Institute, Navi Mumbai, IND; 9 Internal Medicine, University of New Haven, Meriden, USA; 10 Internal Medicine, The Medical Center, Navicent Health, Macon, USA; 11 Internal Medicine, Mercy Catholic Medical Center, Darby, USA; 12 Medicine, Christian Medical College & Hospital, Ludhiana, IND; 13 Epidemiology and Public Health, Icahn School of Medicine at Mount Sinai, New York, USA; 14 Hospital-Based Medicine, Geisinger Commonwealth School of Medicine, Scranton, USA

**Keywords:** acute respiratory distress syndrome, readmission, predictors

## Abstract

Background

The 30-day readmission rates are being used as a quality measure by Centers for Medicare and Medicaid Services (CMS) for specific medical and surgical conditions. Acute respiratory distress syndrome (ARDS) is one of the important causes of morbidity and mortality in the United States (US). The characteristics and predictors of 30-day readmission in ARDS patients in the US are not widely known, which we have depicted in our study.

Objective

The aim of this study is to identify 30-day readmission rates, characteristics, and predictors of ARDS patients using the largest publicly available nationwide database.

Methods

We used the National Readmission Database from the year 2013 to extract the patients with ARDS by primary discharge diagnosis with ICD9-CM codes. All-cause unplanned 30-day readmission rates were calculated for patients admitted between January and November 2013. The independent predictors for unplanned 30-day readmission were identified by survey logistic regression.

Results

After excluding elective readmission, the all-cause unplanned 30-day readmission rate for ARDS patients was 18%. Index admissions readmitted within 30-day had a significantly higher baseline burden of comorbidities with a Charlson Comorbidity Index (CCI) ≥1 as compared to those who were not readmitted within 30 days. In multivariate regression analysis, several predictors associated with 30-day readmission were self-pay/no charge/other (OR 1.19, 95%CI: 1.02-1.38; *p *= 0.02), higher-income class (OR 0.86, 95%CI:0.79-0.99; *p *= 0.03), private insurance (OR 0.81, 95%CI:0.67-0.94; *p *= 0.01), and teaching metropolitan hospital (OR 0.72, 95%CI:0.61-0.94; *p *= 0.01).

Conclusion

The unplanned 30-day readmission rates are higher in ARDS patients in the US. Several modifiable factors such as insurance, socioeconomic status, and hospital type are associated with 30-day readmission among ARDS patients.

## Introduction

Acute respiratory distress syndrome (ARDS) is a syndrome with various etiologies characterized by lung inflammation, increased permeability, pulmonary edema, hypoxemia, and decreased lung compliance [1]. Clinical hallmarks of ARDS are hypoxemia and bilateral radiographic opacities, while the pathological hallmark is diffuse alveolar damage [1]. The clinical features of ARDS usually appear within 6 to 72 hours of an inciting event and worsen rapidly [1]. In a cohort study by Cheung et al., researchers attempted to determine the long-term outcomes of survivors of ARDS and it was determined that ARDS survivors continue to have a functional impairment and compromised health-related quality of life 2 years after discharge from the ICU [2]. Furthermore, patients who require mechanical ventilation in ARDS have a higher severity of illness and a higher number of comorbidities than their non-mechanically ventilated counterparts [3]. These patients also have higher lengths of stay, thus driving up total costs regardless of stable daily costs after the first two days [4-5]. Given the high cost of ARDS management and its life-threatening implications, it is of utmost importance to explore the causes and predictors of 30-day readmission in ARDS patients. These are currently unknown to the US population. The objective of this study was to identify the 30-day readmission rates and modifiable predictors of 30-day readmission in ARDS patients using the largest publicly available nationwide database from the US.

## Materials and methods

Data source

Our retrospective observational study was derived from the subset of the Healthcare Cost and Utilization Project (HCUP) sponsored by the Agency for Healthcare Research and Quality (AHRQ) [6]. The National Readmission Database (NRD) is one of the largest publicly available all-payer inpatient care databases in the United States, including data on approximately 14 million discharges in the year 2013, estimating roughly 36 million weighted discharges from 21 states with reliable, verified linkage numbers [6-7]. NRD represents 49% of total US hospitalizations [7]. Patients were tracked using variable “NRD_Visitlink” used to verify patient linkage number for linking hospital visits for the same patient across hospitals and time between two admissions was calculated by subtracting time variable “NRD_DaysToEvent.” Time to readmission was calculated by subtracting the length of stay (LOS) of primary admissions to time between 2 admissions [8-9]. National estimates are produced using sampling weights provided by the NRD. The details regarding the NRD data are available online [7-8].

Study population and design

**Figure 1:**
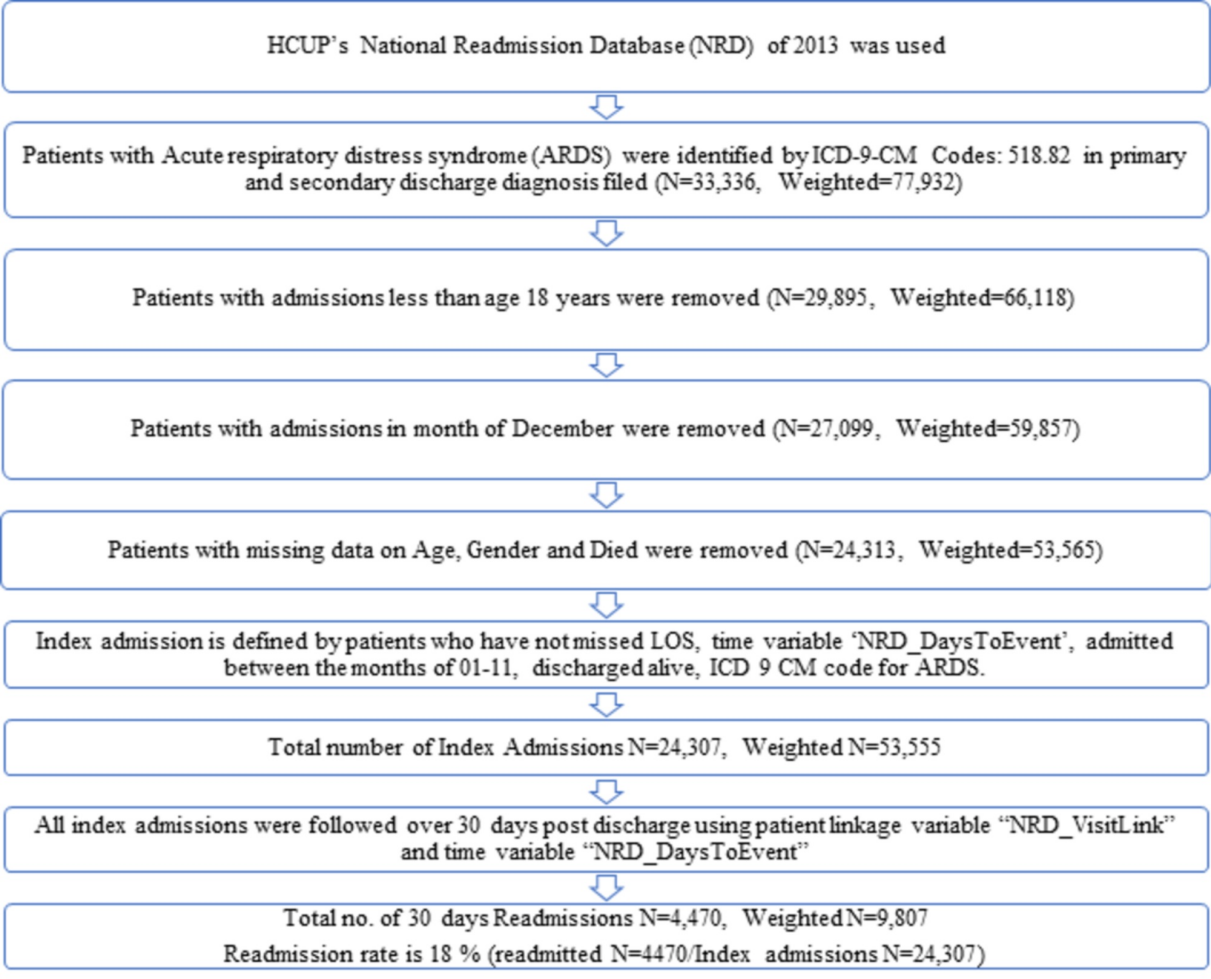
Sequential derivation of study population

All variables were identified using the International Classification of Diseases, Ninth Revision, Clinical Modification (ICD-9-CM) Volume 3 diagnosis codes. We queried the NRD database using the presence of ICD-9-CM diagnosis code of 518.82 for ARDS in primary or secondary diagnostic fields which only includes ARDS related to non-trauma or surgical causes. We identified 24,307 ARDS patients (weighted N= 53,555) after excluding patients with missing data for age or gender. We also excluded procedures done in the month of December, as we did not have follow-up data for the same, data points with LOS of 0 days were also excluded. Figure 1 shows the sequential derivation of the study cohort. Patients who were readmitted to the hospital within 30 days within the same calendar year were further evaluated.

Outcome variables

The primary outcome was 30-day readmission rate. Secondary outcomes included predictors of readmission in ARDS patients. NRD variables were used to identify patients’ demographic characteristics including age, gender, hospital characteristics such as bed size and teaching status, and other patient-specific characteristics including median household income category for patient’s zip code, primary payer, admission type, admission day, and discharge disposition [8]. We identified various co-morbid conditions as it is provided by AHRQ co-morbidity (cm_) measures with the database. Additionally, Deyo’s modification of the Charlson comorbidity index (CCI) was used to define the severity of co-morbid conditions [10-12]. Deyo modification of Charlson co-morbidity index (CCI), which contains 17 co-morbid conditions was used as a measure of co-morbidity burden [10]. With score ranges from zero to 33, a higher score means a greater burden of co-morbid diseases [10].

Statistical analysis

We utilized Statistical Analysis Software (SAS) 9.4 (SAS Institute, Cary, NC, USA) for all analyses. Survey procedures were implemented to adjust for stratified cluster design of NRD with DOMAIN, STRATA, CLUSTER, and WEIGHT statements [13]. We used the *X*^2^ test for categorical variables, t-test for normally distributed continuous variables, and the Wilcoxon rank-sum test for non-normally distributed continuous variables to compare the baseline characteristics of the study population. P-values less than 0.05 were considered significant. The independent predictors of unplanned 30-day readmission were identified by multivariate logistic regression adjusting for stratified cluster design of NRD. Multivariate models for readmission included hospital-level variables such as age, gender, admission type (elective vs. non-elective), median household income (higher income quartile vs. lowest income quartile), the primary payer (private insurance vs. self-pay vs. Medicaid/Medicare). The multivariate model for readmission was run only on patients who survived index admission. All interactions were thoroughly tested. Few observations and response variables were deleted due to missing or invalid values for its explanatory, frequency, weight, strata, or cluster variables.

## Results

Readmission rate

There were 24,307 (weighted N = 53,555) index admissions for ARDS in the US during the study period out of which, 4,470 (weighted N = 9,807) patients were readmitted within 30 days, which inferred the readmission rate at 18% (Figure 1).

Baseline characteristics of index admissions stratified by presence of 30-day readmission

Index admissions who were readmitted within 30-day had a significantly higher baseline burden of comorbidities with a CCI ≥1 (87% vs. 81%; *p* < 0.001) as compared to those who did had 30-day readmission. Similarly, comorbidities like hypertension (63% vs 60%; *p* < 0.001), electrolyte imbalance (50% vs 43%; *p* < 0.001), renal failure (28% vs. 20%; *p* < 0.001), and diabetes mellitus (27% vs 24%; *p* < 0.001) were higher among those index admissions who had 30-day readmission. The index admissions that had readmission were more likely to be from large bed size hospital (67% vs. 64%; *p* < 0.001), were admitted non-electively (93% vs. 90%; p <0.001) and had Medicare as an insurance (67% vs. 63%; *p* < 0.001). Additionally, an index admission with readmission had higher proportion of discharge disposition to facility (32% vs. 26%; *p* < 0.001) and LOS ≥7 days (48% vs. 40%; *p* < 0.001) than those without readmits. Other characteristics, including age, gender, and hospital teaching status were approximately evenly distributed among both groups. Another detailed distribution of patient and hospital-level characteristics has been depicted in Table 1.

**Table 1:** Baseline characteristics of index admissions stratified by presence of 30-day readmission z - Charlson/Deyo co-morbidity index (CCI) was calculated as per Deyo classiﬁcation. * Variables are AHRQ co-morbidity (cm_) measures. x- Represents a quartile classiﬁcation of the estimated median household income of residents in the patient’s ZIP Code, derived from ZIP Code-demographic data obtained from Claritas. The quartiles are identiﬁed by values of 1 to 4, indicating the poorest to wealthiest populations. Because these estimates are updated annually, the value ranges vary by year. http://www.hcupus.ahrq.gov/db/vars/zipinc_qrtl/nisnote.jsp. # -HMO: Health Maintenance Organization. {- The bed size cutoff points divided into small, medium, and large have been done so that approximately one-thirdof the hospitals in a given region, location, and teaching status combination would fall within each bed size category. State and County QuickFacts. Washington, DC: US Census Bureau; 2012.

	Index Admission	No Readmission	Readmission	p value
Total population (unweighted)	24,307	19,837	4,470	
Total population (weighted)	53,555	43,748	9,807	
Patient level variables				
Age (%)				0.08
≥85	14	14	13	
65-84	29	29	30	
45-64	39	40	40	
18-44	16	17	17	
Gender (%)				0.09
Female	52	53	52	
Male	47	47	48	
CCI^z^ (%)				.001
0	18	19	13	
1-3	56	56	53	
4-6	19	18	26	
7-9	5	5	7	
10-12	1	1	1	
13-15	0.05	0.05	0.05	
≥16	0.06	0.00	0.01	
Length of stay (%)				.001
1-3	29	31	25	
4-6	28	28	26	
7-9	16	16	17	
10-12	8	8	9	
13-15	5	5	7	
≥16	12	11	15	
Comorbidities (%) *				
Drug abuse	4	4	5	0.08
Hypertension	61	60	63	.001
Hypothyroidism	15	15	16	0.004
Liver disease	4	4	5	.001
Fluid and electrolyte disorders	44	43	50	.001
Metastatic cancer	4	3	5	0.003
Neurological disorders	12	12	13	0.12
Obesity	16	17	15	0.07
Peripheral vascular disorders	8	8	10	.001
Psychoses	5	5	7	0.001
Pulmonary circulation disorders	5	6	6	0.3
Renal Failure	22	20	28	.001
Valvular disease	7	7	8	0.28
Alcohol abuse	5	5	5	0.26
Rheumatoid arthritis/ collagen vascular diseases	4	4	4	0.58
Chronic blood loss anemia	1	1	2	0.12
Congestive heart failure	18	17	22	.001
Chronic pulmonary disease	19	19	20	0.15
Depression	13	13	13	0.99
Diabetes, uncomplicated	25	24	27	0.002
Diabetes with chronic complications	7	7	10	.001
Median household income (%) ^x ^				0.1
76-100^th^	27	26	28	
51-75^th^	26	26	24	
26-50^th^	25	25	25	
0-25^th^	21	22	21	
Primary Payer (%)				.001
Self-pay/no charge/other	63	63	67	
Private including HMO^#^	11	10	13	
Medicaid	18	19	15	
Medicare	8	8	5	
Hospital bed size (%) ^{^				0.02
Large	11	11	10	
Medium	24	25	23	
Small	65	64	67	
Hospital teaching status (%)				0.05
Non-metropolitan hospital	32	32	32	
Metropolitan teaching	58	58	59	
Metropolitan non-teaching	10	10	9	
Admission type (%)				
Elective	91	90	93	
Non elective	9	10	7	
Disposition (%)				.001
Against medical advice	50	52	42	
Facilities	22	21	24	
Home health care	27	26	32	
Home	1	1	2	

Predictors of 30-day readmission

From our analysis, several predictors associated with increased 30-day readmission were higher CCI (OR for ≥3 1.147, 95%CI: 1.065-1.236; *p *< 0.001), self-pay/no charge/other (OR 1.19, 95%CI: 1.02-1.38; *p *= 0.02), discharge disposition against medical advice (OR 1.25, 95%CI:1.12-1.39; *p *< 0.001), and discharge disposition to facility (OR 1.28, 95%CI 1.15-1.43; *p *< 0.001). Meanwhile, we also found several predictors associated with reduced odds for 30-day readmission: higher income class (OR 0.86, 95%CI:0.79-0.99; *p *= 0.03), private insurance (OR 0.81, 95% CI:0.67-0.94; *p *= 0.01), and teaching metropolitan hospital (OR 0.72, 95% CI:0.61-0.94; *p *= 0.01). On the other hand, several characteristics were not found to be associated with increased or decreased readmission (Table 2).

**Table 2:** Multivariate analysis in 30-day readmission in ARDS patients z - Charlson/Deyo co-morbidity index (CCI) was calculated as per Deyo classiﬁcation. *Variables are AHRQ co-morbidity (cm_) measures. x- Represents a quartile classiﬁcation of the estimated median household income of residents in the patient’s ZIP Code, derived from ZIP Code-demographic data obtained from Claritas. The quartiles are identiﬁed by values of 1 to 4, indicating the poorest to wealthiest populations. Because these estimates are updated annually, the value ranges vary by year. http://www.hcupus.ahrq.gov/db/vars/zipinc_qrtl/nisnote.jsp. # -HMO: Health Maintenance Organization. {- The bed size cutoff points divided into small, medium, and large have been done so that approximately one-third of the hospitals in a given region, location, and teaching status combination would fall within each bed size category. State and County QuickFacts. Washington, DC: US Census Bureau; 2012. ARDS, acute respiratory distress syndrome

Predictors	Odds Ratio	95% CI	p value
Age			
≥85	0.97	0.81-1.15	0.67
65-84	0.75	0.61-0.92	0.001
45-64	0.69	0.55-0.88	0.0002
18-44	Ref		
Gender			
Female	0.96	0.88-1.05	0.43
Male	Ref		
CCI^z^			
≥16	1.29	1.14-1.46	.001
13-15	1.39	1.14-1.70	.001
10-12	1.24	0.87-1.76	0.23
7-9	0.61	0.08-4.73	0.64
4-6	1.98	0.14-28.82	0.62
0-3	Ref		
Length of stay			
≥16	1.16	1.03-1.31	0.01
13-15	1.26	1.11-1.44	0.0003
10-12	1.27	1.09-1.45	0.003
7-9	1.51	1.26-1.78	.001
4-6	1.39	1.19-1.62	.001
1-3	Ref		
Comorbidities*			
Drug abuse	1.23	0.96-1.57	0.11
Hypertension	1.04	0.94-1.14	0.49
Hypothyroidism	1.14	1.02-1.27	0.02
Liver disease	1.19	0.99-1.42	0.06
Fluid and electrolyte disorders	1.09	1.00-1.19	0.16
Metastatic cancer	1.09	0.85-1.38	0.3
Neurological disorders	1.03	0.88-1.19	0.49
Obesity	1.01	0.89-1.14	0.73
Peripheral vascular disorders	0.82	0.73-0.93	0.002
Psychoses	1.14	0.95-1.37	0.88
Pulmonary circulation disorders	0.95	0.79-1.13	0.16
Renal Failure	1.15	1.00-1.29	0.54
Peptic ulcer disease,	3.99	1.30-12.38	0.01
excluding bleeding			
Valvular disease	0.92	0.78-1.06	0.2
Alcohol abuse	0.99	0.79-1.12	0.21
Rheumatoid arthritis/	0.96	0.79-1.15	0.85
collagen vascular diseases			
Chronic blood loss anemia	1.18	0.88-1.57	0.65
Congestive heart failure	1.13	1.01-1.25	0.27
Chronic pulmonary disease	0.95	0.86-1.05	0.3
Depression	0.96	0.85-1.05	0.32
Diabetes,	1	0.91-1.10	0.71
uncomplicated			
Diabetes with chronic complications	1.15	1.00-1.31	0.99
Median household income ^x^			
76-100^th^	0.86	0.79-0.99	0.03
51-75^th^	0.97	0.86-1.09	0.62
26-50^th^	1.19	1.02-1.38	0.12
0-25^th^	Ref		
Primary Payer			
Self-pay/no charge/other	1.19	1.02-1.38	0.02
Private including HMO^#^	0.81	0.67-0.94	0.01
Medicaid	0.59	0.47-0.74	.001
Medicare	Ref		
Hospital bed size ^{^			
Large	1.04	0.86-1.25	0.69
Medium	0.96	0.87-1.05	0.13
Small	Ref		
Hospital teaching status			
Non-metropolitan hospital	0.85	0.72-1.01	0.36
Metropolitan teaching	0.72	0.61-0.84	0.02
Metropolitan non-teaching	Ref		
Admission type			
Elective	0.72	0.61-0.84	.001
Non elective	Ref		
Admission day			
Weekend Admission	0.98	0.88-1.09	0.69
Weekday	Ref		
Disposition			
Against medical advise	1.25	1.12-1.39	<0.001
Facilities	1.28	1.15-1.43	<0.001
Home health care	2.42	1.64-3.55	<0.001
Home	Ref		

## Discussion

We report contemporary data from an NRD on readmission of patients with ARDS. Our study reports an all-cause unplanned 30-day readmission rate of 18% in ARDS patients. A Canadian study on long-term outcomes of ARDS has an estimated 39% readmission in the first 2 years after the discharge [2]. This study was from four academic tertiary care ICUs from a city [2]. However, our study is based on a large administrative database, which can be weighted to produce national estimates. Our study also highlights that ARDS patients who got readmitted in 30 days had a higher burden of co-morbidities especially hypertension, electrolyte disorders, and diabetes. Pan et al. found that higher CCI is associated with the increased risk of admission which we have also repopulated in our study, CCI ≥3 (OR 1.147, 95%CI: 1.065-1.236; p<0.001) [14]. Furthermore, we found several other factors associated with increased 30-day readmission. We observed that patients who leave against medical advice showed a higher risk for unplanned 30-day readmission for ARDS. Furthermore, discharge to the facility was also a significant predictor of readmission in our study, which was also shown by Pan et al. [14]. This could be because the patients being discharged to facilities are likely sicker with a higher number of co-morbidities or newly acquired morbidity or system failure due to the effect of ARDS [15]. Further, we observed that self-pay/No charge/other had higher odds of unplanned 30-day readmission, and patients with the higher socioeconomic income class have the reduced odds of 30-day readmission, which signifies that socioeconomic factors might have played a role in leading to readmissions in the ARDS population. Meanwhile, Ferro EG et al. showed the percentage of readmissions was lower among the patients with private insurance in all-cause index hospitalizations [16]. Interestingly, we also observed a similar phenomenon in ARDS index hospitalizations. It is known that Medicaid patients have greater difficulty obtaining appointments in comparison to privately insured patients [17]. We postulate that privately insured patients have a better follow-up compared to others and hence, they had lower readmission rates in our study. We also found reduced odds of readmission in the patients at teaching metropolitan hospitals, which can be attributed to the advanced medical care and better implementation of the quality improvement programs in the academic settings. Khaksari BJ et al. showed no association between weekend admission and 30-day readmission, which was seen in our study as well [18].

Our study has several limitations. First, our study is a retrospective study which makes it subjectable to selection bias. Secondly, the selection of samples relies on the sensitivity and specificity of ICD-9-CM codes which could have confounded our results. Also, the NRD database does not contain information on the long-term follow-up of the patients. Our study also lacks data on ARDS patients discharged to home hospice who are not at risk of being readmitted, which might result in an underestimation of the readmission rate. Despite its limitations, our study has several strengths. Our study is the first nationwide study looking at readmission rates and predictors of ARDS. Our sample size most closely represents the standardized U.S. population. Targets for future interventions should be focused on better risk stratification and controlling the predictors that aggravate the readmission rates of ARDS, which we were able to identify successfully.

## Conclusions

The unplanned 30-day readmission rates are higher for ARDS patients in the US. There are several modifiable factors such as type of insurance, socioeconomic status, and hospital type associated with 30-day readmission among ARDS patients. Further studies are needed to identify preventable readmissions, aim to modify the predictors, and develop a strategic approach to reduce the burden of readmissions in patients admitted with ARDS.
